# The multifaceted nature of diabetic erectile dysfunction: uncovering the intricate mechanisms and treatment strategies

**DOI:** 10.3389/fendo.2024.1460033

**Published:** 2024-11-08

**Authors:** Jianxiong Ma, Yihao Chen, Yuhe Si, Jiahua Qian, Chenxi Wang, Juan Jin, Qiang He

**Affiliations:** ^1^ The First Affiliated Hospital of Zhejiang Chinese Medical University (Zhejiang Provincial Hospital of Chinese Medicine), Hangzhou, Zhejiang, China; ^2^ The First Clinical Medical College, Zhejiang Chinese Medical University, Hangzhou, Zhejiang, China; ^3^ The Second Clinical Medical College, Zhejiang Chinese Medical University, Hangzhou, Zhejiang, China

**Keywords:** diabetes mellitus erectile dysfunction, pathological mechanisms, treatment strategies, interdisciplinary approach, clinical implications

## Abstract

**Background:**

One of the most common complications of diabetes mellitus is diabetic erectile dysfunction (DMED), a condition that has grown more common in recent years and has a significant impact on patients’ daily lives. The complicated pathophysiological changes of DMED, involving vascular, neurological, muscular, and endocrine variables, have not been well addressed by any one treatment technique, and no widely approved treatment strategy has been developed.

**Aim:**

The objective of this study was to thoroughly examine the complex nature of the pathogenic mechanism of DMED and discover new therapeutic approaches that could improve DMED symptoms.

**Methods:**

Studies and review articles from the past 10 years were considered.

**Results:**

The pathogenesis of DMED encompasses vascular dysfunction, endothelial cell damage, cavernous smooth muscle defects, neurological dysfunction, endocrine/metabolic factors, leukomalacia fibrosis, and psychosocial factors, elucidating complex interplay among the mechanisms underlying DMED. It underscores the need of integrating traditional herbal medicine, energy-based medicine treatments, and advanced techniques like stem cell and gene therapy to enhance therapeutic outcomes. Furthermore, it expresses optimism on the therapeutic potential of new nanobiomaterials in DMED.

**Conclusion:**

Through integrating a complete description of DMED etiology and current therapy methods, this work offers a helpful resource for researchers, doctors, and patients dealing with this difficult condition.

## Background

Diabetes Mellitus Erectile Dysfunction (DMED) is a prevalent consequence of diabetes mellitus (DM) that specifically impacts the corpus cavernosum tissue responsible for erectile function. Studies have shown that DMED is prevalent in men with type 1, type 2, and both types of diabetes, with an overall prevalence of 52.5% (1). The incidence of erectile dysfunction (ED) is greater in males with compromised fasting blood glucose and impaired glucose tolerance in comparison to those with normal blood glucose levels (29.2% and 33.2% vs. 14.8%) (2). On the other hand, diabetes is a worldwide health issue, impacting 536.6 million individuals in 2021 and expected to rise to 783.2 million prior to 2045 (3). An analysis of diabetes in China has shown that the occurrence of diabetes in the adult population has increased from 0.67% to 11.2% over the last forty years, affecting almost 140.9 million people (4). An analysis of diabetes in China has shown that the occurrence of diabetes in the adult population has increased from 0.67% to 11.2% over the last forty years, affecting almost 140.9 million people.

Predicting and evaluating illness risk through the use of genetic databases has been the focus of research in recent years. The unique capacity of these databases lies in their ability to efficiently reduce the influence of complicating factors and clarify causal connections. An extensive genome-wide association study included 223,805 Europeans presented evidence that type 2 diabetes is an independent risk factor for erectile dysfunction (ED), therefore reinforcing the robust link between DM and ED (5). Through several processes, including persistent hyperglycemia, variable blood sugar levels, and aberrant glucose metabolism byproducts, diabetes adversely impacts the erectile organ. These elements can hinder the flow of blood, disturb the functioning of blood vessels, degrade the integrity of nerves, weaken the structures of muscles, and disturb the essential endocrine processes involved in the erection mechanism. In our prior study, we investigated the development of DMED and explored the potential of natural drug therapy. Our findings indicate that a combination of leech centipede drugs may enhance the erectile function of diabetic rats by suppressing endothelial cell death and penile fibrosis (6).

**Figure 1:**
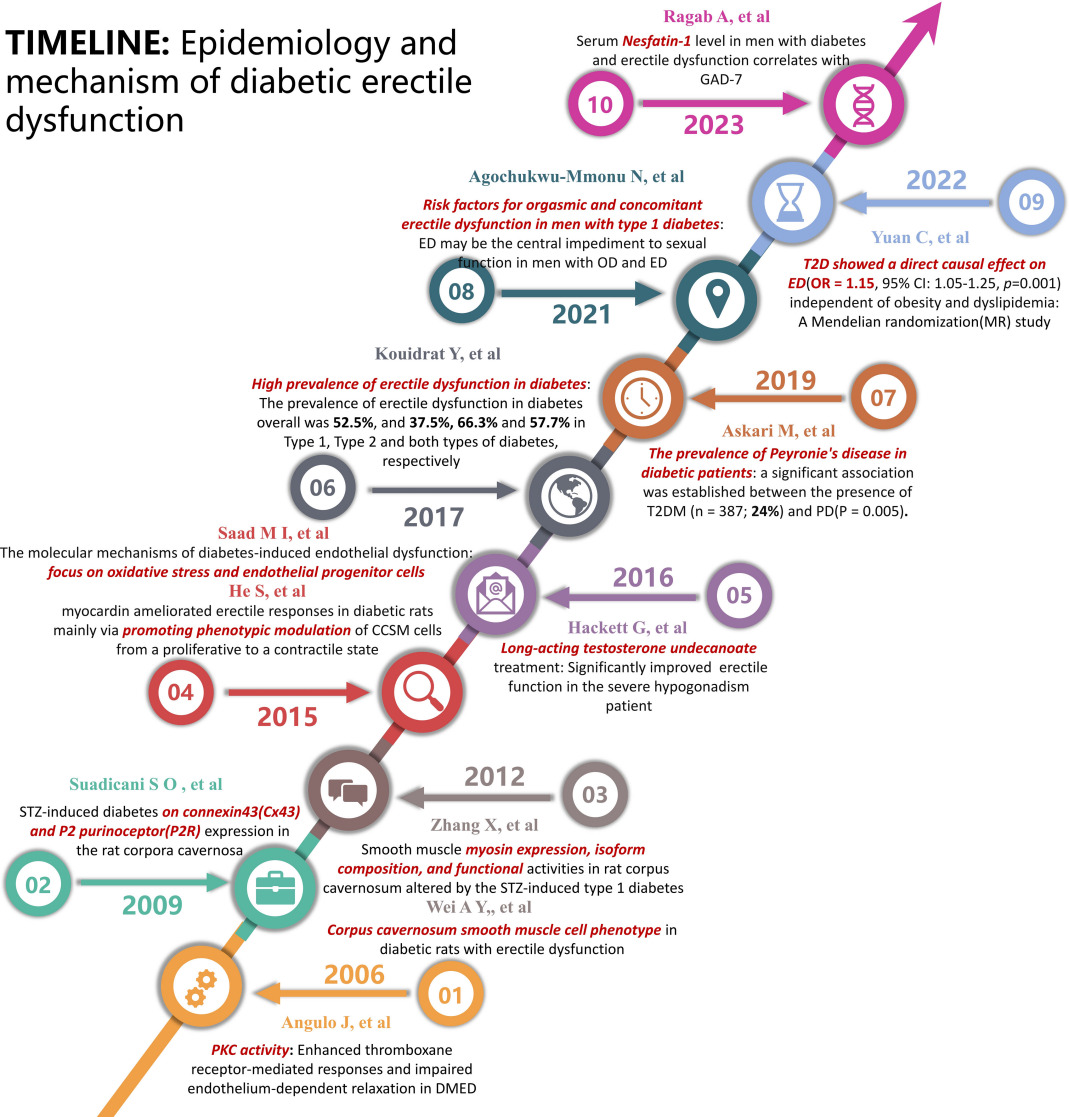
Timeline of Epidemiology and mechanism of diabetic erectile dysfunction.

**Figure 2:**
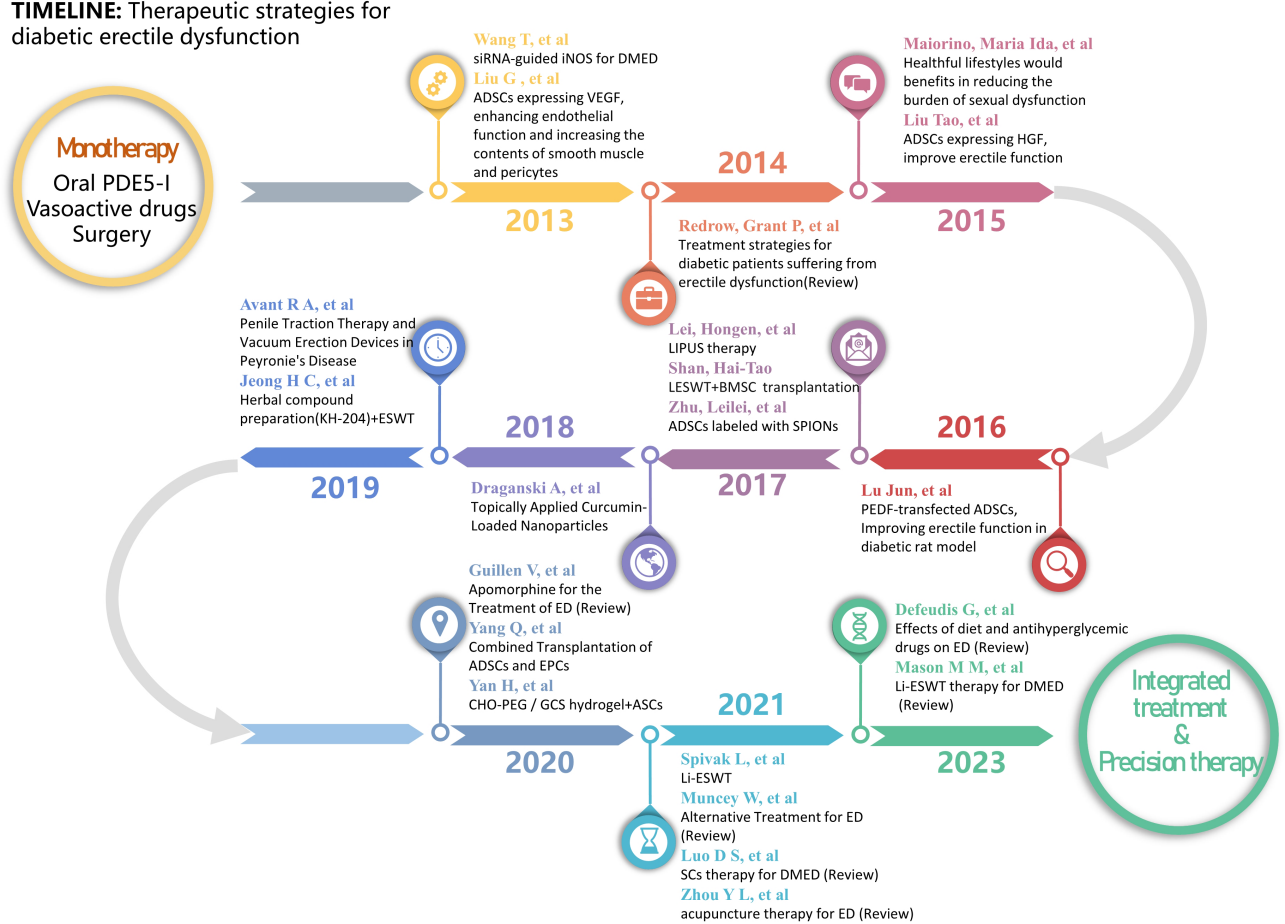
Timeline of Therapeutic strategies for diabetic erectile dysfunction.

Nevertheless, recent studies on the development of DMED evidence the presence of several mechanisms of injury. The mostly treatment-resistant character of DMED in clinical settings is mostly attributed to the lack of specific therapeutic approaches. Consequently, the study of the development of DMED and the exploration of new treatment methods have continuously been the main areas of research in the field of andrology. This paper presents a succinct summary of the progress achieved in this field throughout the past ten years. A diagram illustrating the study development on the epidemiology and mechanism of DMED is presented in **Figure 1**. The state of research on DMED therapy approaches is illustrated in **Figure 2**.

## Physiological mechanism of erection

Sexual erection is a complex physiological process that encompasses several systems such as psychology, endocrine function, blood vessels, and nerves. Initiation of sexual desire can occur through audio-visual, tactile, or imaginative stimulus, which then stimulates impulses to the penile cavernous body through the lower hypothalamus center or sacral cord. The activation of guanylate cyclase (GC) through the GTP/cGMP pathway is attributed to the neurotransmitter Nitric Oxide (NO). The outcome of this phenomenon is an elevation in the concentrations of cyclic guanosine monophosphate (cGMP) in the smooth muscle cells of the cavernous body. Augmented arterial blood flow, hyperemia, and expansion of the penile cavernous body are facilitated by the relaxation of the trabecular smooth muscle induced by the rise in cGMP levels (7). The distended corpus cavernosum exerts pressure on the minor veins located beneath the tunica albuginea, therefore blocking the route of venous outflow. Moreover, the contraction of the pelvic floor muscles might enhance the compression of the corpus cavernosum, therefore increasing its size and structural integrity to aid in achieving an erection (8, 9).

### Mechanisms of erectile dysfunction in diabetes: a pathological analysis

**Figure 3:**
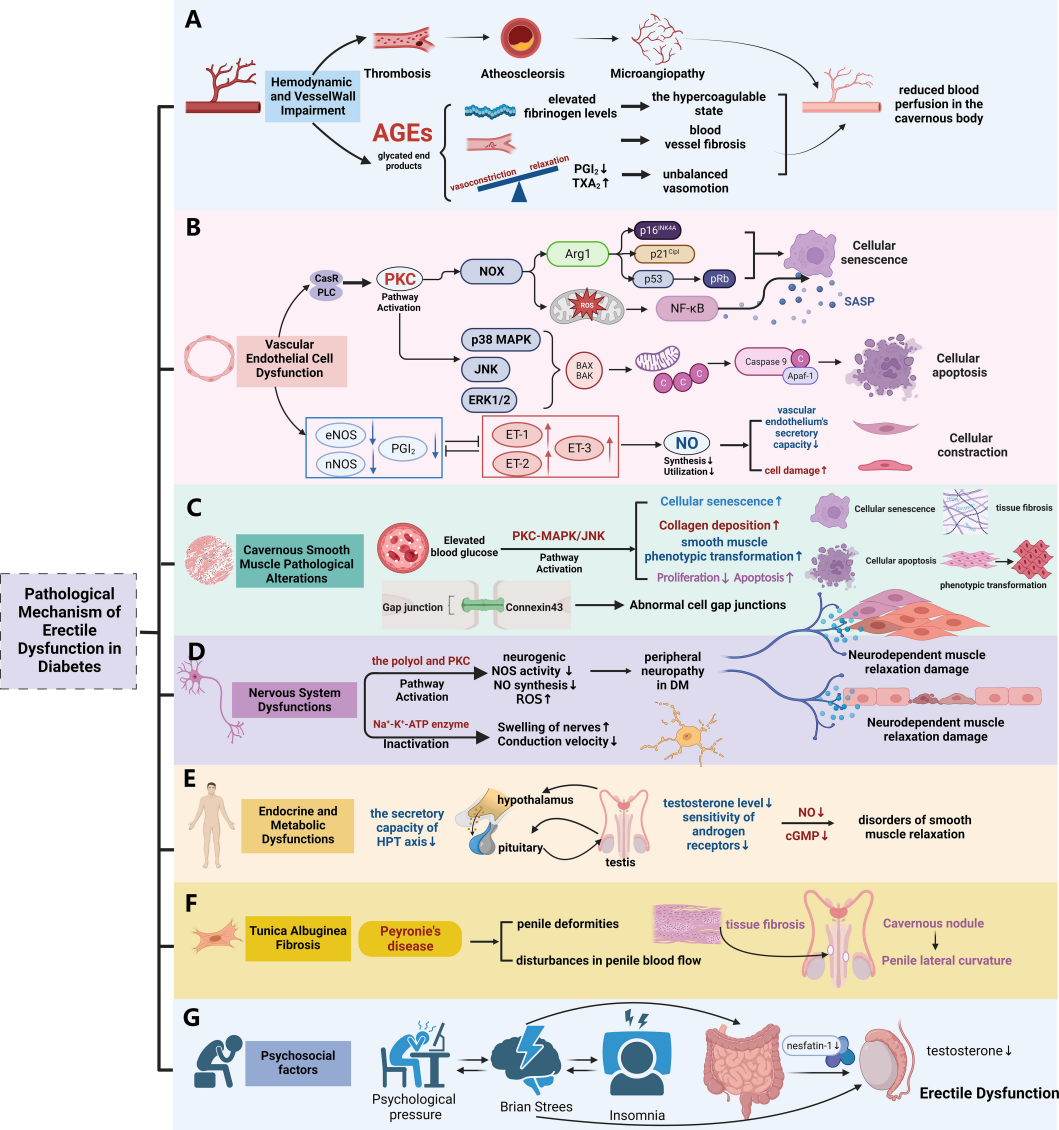
The pathological mechanism of diabetic erectile dysfunction. **(A)** The pathological mechanism of erectile dysfunction mediated by hemodynamic changes and Vessel Wall Impairment in diabetes; **(B)** The pathological mechanism of erectile dysfunction mediated by vascular endothelial dysfunction in diabetes; **(C)** The pathological mechanism of erectile dysfunction mediated by Cavernous Smooth Muscle Pathological Alterations in diabetes; **(D)** The pathological mechanism of erectile dysfunction mediated by Nervous System Dysfunctions in diabetes; **(E)** The pathological mechanism of erectile dysfunction mediated by Endocrine and Metabolic Dysfunctions in diabetes; **(F)** The pathological mechanism of erectile dysfunction mediated by Tunica Albuginea Fibrosis in diabetes; **(G)** Influence of psychosocial factors on erectile function in patients with diabetes. (Used Abbreviations and symbols in figure: AGEs, advanced glycation end products; PGI2, prostaglandin I2; TXA2, thromboxane; CaSR, calcium-sensing receptor; PLC, phospholipase C; PKC, protein kinase C; NOX, coenzyme oxidase; Arg1, arginase 1; ROS, reactive oxygen species; SASP, Senescent Associated Secretory Phenotypes; MAPK, mitogen-activated protein kinase; ET-1, endothelin; DM, diabetes mellitus; HPT axis, hypothalamus-pituitary-testis axis; cGMP, cyclic guanosine monophosphate) Figure created by BioRender.com.

Diabetes has the potential to impair male erectile function by disabling blood vessels, nerves, gonadal axis, muscles, and several other organs and tissues. This damage can result from disturbances to many pathways, including the polyol pathway, protein kinase C (PKC) pathway, and others (as shown in **Figure 3**).

### Hemodynamic and vessel wall impairment

Research has demonstrated that individuals with diabetes may have increased viscosity of whole blood and abnormal aggregation of red blood cells. This phenomenon could be attributed to an over activation of platelets and an increased expression of genes involved in cell surface adhesion (10, 11). Furthermore, individuals with type 2 diabetes may exhibit increased concentrations of fibrinogen, the predominant clotting factor in the plasma. Additionally, in diabetic individuals, fibrinogen can stimulate thrombin activation, resulting in the production of insoluble fibrin and ultimately leading to the development of blood clots (12, 13). This mechanism hinders the circulation of blood within the cavernous corpus.

Furthermore, increased glucose metabolism can also impact the artery wall. Advanced glycation end products (AGEs) can induce fibrosis of blood vessels, causing a decrease in the flexibility and compliance of cavernous blood vessels. This ultimately leads to a reduction in blood flow to the cavernous body (14–16). Diabetes also disrupts the equilibrium between vasoconstriction and relaxation receptors, such as prostaglandin I2 (PGI2) and thromboxane (TXA2) (17, 18). Diabetes also disrupts the equilibrium between vasoconstriction and relaxation receptors, such as prostaglandin I2 (PGI2) and thromboxane (TXA2) (19).

### Vascular endothelial cell dysfunction

Specialized architecture characterizes the penis, a vascular tissue that includes endothelial cells (ECs) as its innermost layer of blood vessels. Endothelial cells (ECs) play an active role in maintaining the balance of blood vessels and the smooth muscles in their vicinity. Recent research has revealed four main routes that contribute to the damage and dysfunction of endothelial cells in the hyperglycemic setting of diabetic mellitus (DM): the AGEs pathway, polyol pathway, hexosamine pathway, and PKC pathway (20, 21). The investigation of hyperglycemia-triggered vascular endothelial dysfunction in DM includes a primary focus on the PKC pathway. The conventional mechanism of PKC activation is the connection of the calcium-sensing receptor (CaSR) with phospholipase C (PLC) driven by the high-glucose environment. Following this interaction, phosphatidylinositol-4,5-bisphosphate (PIP2) is broken down, resulting in the formation of diacylglycerol (DAG) and inositol triphosphate (IP3). Later interaction of IP3 with very specific receptors on the endoplasmic reticulum and nucleus triggers the activation of calcium ion channels, so enabling the liberation of calcium ions from the nucleus into the cytoplasm. The activation of PKC proteins situated on the inner side of the plasma membrane is facilitated by this cascade in conjunction with DAG (22, 23).

Following the activation of PKC, two stages of advancement occurred. On one side, it enhances the generation of oxygen free radicals by inhibiting the activity of reduced coenzyme oxidase (NOX), therefore decreasing the accessibility of nitric oxide (NO). The secretory capacity of the vascular endothelium is influenced by the elevation of endothelin (ET-1) and vascular endothelial growth factor (VEGF) levels induced by this process. This sequence results in the impairment of endothelium-dependent relaxation of vascular and muscle cells (24). O Conversely, the regular secretion and function of endothelial-derived nitric oxide (NO) contributes to the preservation of the relaxation of cavernosal smooth muscle, vascular smooth muscle cells, and the physiological basis of erection in the penis. In addition, the activation of NOX can enhance the expression of its focal protein, arginase 1 (Arg1), and the production of reactive oxygen species (ROS) (25). This, in turn, stimulates an increase in the expression of p16[Ink4a], p21[Cip1], and p53, so facilitating cellular senescence. In contrast, the elimination of the Arg1 gene or its pharmacological suppression effectively inhibits the aging of endothelium cells in diabetic mice (26).

Furthermore, PKC has the ability to initiate the consecutive activation of mitogen-activated protein kinase (MAPK), resulting in the activation of amino-terminal kinases like JNK, ERK, and p38. This sequence of events leads to cellular injury, initiates preprogrammed cell death, increases the expression of pro-apoptotic proteins and Bak, and decreases the expression of the anti-apoptotic protein Bcl-2. This process promotes the liberation of cytochrome C and mitochondrial DNA from the mitochondrial membrane into the cytoplasm, together with an upregulation of the pro-apoptotic protein Bax (27). Conversely, the expression of the anti-apoptotic protein Bcl-2 decreases, which in turn promotes the release of cytochrome C from the inner mitochondrial membrane into the cytoplasm. Consequently, the development of apoptotic complexes is triggered, leading to the predetermined death of vascular endothelial cells. Profound mitochondrial failure produces a substantial amount of reactive oxygen species (ROS) that stimulate NF-κB. Upon activation, NF-κB dissociates from inhibitory proteins, therefore enabling its action on the κB configuration of DNA. Moreover, it facilitates the release of many Senescent Associated Secretory Phenotypes (SASP), therefore worsening the process of endothelial cell senescence (28). In conclusion, hyperglycemia in DM patients can induce endothelial cell dysfunction, leading to diminished synthesis and utilization of nitric oxide, consequently fostering the onset and progression of DMED (29).

### Cavernous smooth muscle pathological alterations

Cavernous smooth muscle cells (CCSMC) play a vital role in penile erection, constituting 42-50% of the cavernosal cell population. Their relaxation is essential for prompt initiation and sustained maintenance of an erection (30–33). However, increased blood glucose levels can decrease the production of smooth muscle gap junction proteins and change the ratio of myosin subtypes in muscle cells (34). This can result in damage to elastic fibers, atrophy of smooth muscle, excessive buildup of collagen, and a modification in the phenotypic transition of smooth muscle (35–37). When smooth muscle cells undergo a transition from a contractile state to a synthetic state, characterized by enhanced proliferation and migration, the levels of α-smooth muscle actin (α-SMA) and desmin are reduced, but osteopontin (OPN) is increased (38). Consequently, the contraction of cells is disturbed, which can have a substantial impact on the development of vascular complications and erectile dysfunction in rats with early-onset diabetes (39).

Studies have shown that high glucose levels activate the PKC-MAPK/JNK signaling pathway, which contributes to the phenotypic transformation of smooth muscle cells (40). In diabetic patients, hyperactivation of PKC causes increased contractility and reduced endothelium-dependent relaxation in human CCSMCs (41). It also plays a crucial role in mediating smooth muscle cell senescence, which contributes to vascular aging, vascular calcification, and atherosclerosis (42). By inhibiting PKC upstream signaling, particularly DAG, it is possible to reduce the expression of the MAPK/ERK1/2 and PI3K/Akt signaling pathways, which in turn, mitigates the migration and mitosis of vascular smooth muscle cells in models of atherosclerosis and restenosis (43).

Previous research has shown that CCSMCs in rats with diabetic ED undergo phenotypic transformation. When myocardin gene transfection was performed in these rats, CCSMCs transitioned from a synthetic to a contractile phenotype. This transition was accompanied by up-regulated expressions of marker proteins for contractile smooth muscle cells, namely α-SMA and desmin. On the other hand, the expressions of OPN and Vimentin—marker proteins associated with synthetic smooth muscle cells—were down-regulated. It is noteworthy that this transformation led to a substantial improvement in erectile function (44, 45).

Studies have indicated that downregulation of the CAT/3-MST and DAO/3-MST pathways, coupled with reduced activity of CBS and CSE, leads to a significant decrease in endogenous hydrogen sulfide (H_2_S) production in the penile tissue of patients with diabetic mellitus erectile dysfunction (DMED). H_2_S can activate potassium channels on smooth muscle cells, increasing intracellular potassium ion concentration, which results in membrane hyperpolarization. This hyperpolarization inhibits calcium ion influx, reduces actin-myosin sliding, and induces relaxation of corporal smooth muscle cells (CCSMCs), contributing to penile erection. Consequently, the reduction in H_2_S levels is also a significant factor in the development of erectile dysfunction (ED) (46).

### Nervous system dysfunctions

The neurological system regulates the mechanism of regular penile erection. Prolonged hyperglycemia can result in impaired glucose metabolism, insulin pathways, oxygen free radical levels, mitochondrial activity, and microcirculation, therefore causing peripheral neuropathy and degeneration of pudendal sensory neurons (47, 48) and a decrease in sexual stimulus conduction (49). In addition to sensory nerves, degeneration of efferent nerves can also cause a decrease or halt in parasympathetic nerve activity, which regulates the relaxation of cavernous smooth muscles. This can lead to impaired neurogenic NOS activity and reduced NO synthesis (50). In addition to sensory nerves, degeneration of efferent nerves can also cause a decrease or halt in parasympathetic nerve activity, which regulates the relaxation of cavernous smooth muscles. This can lead to impaired neurogenic NOS activity and reduced NO synthesis (51). The over conversion of sorbitol in the polyol pathway is a chronic problem that leads to neurotrophic diseases, producing nerve swelling, distortion, or necrosis and causing long-lasting inflammatory alterations in the peripheral nerves. Moreover, this buildup can have a detrimental impact on the NO-regulated GTP/cGMP pathway, thus resulting in erectile dysfunction (52). The activation of the PKC pathway can stimulate the generation of reactive oxygen species (ROS), leading to nerve fiber damage and subsequent pathological alterations (53). High sugar levels can impede the uptake of inositol by nerve tissue, therefore reducing the activity of Na+-K+ -ATPase, leading to neuron swelling and reduced conduction velocity (54). Hyperglycemia-induced autonomic nerve injury can result in nerve dysfunction and damage to some autonomic nerves, including adrenergic and cholinergic nerves, which innervate penile blood vessels. The asymmetry in arteriovenous contraction during penile erection might result in penile venous leakage, ultimately leading to ED (55).

### Endocrine and metabolic dysfunctions

Optimal release and effective use of male hormones are crucial in promoting sexual desire and preserving male erectile function. Nevertheless, an extended period of elevated glucose levels, as shown in diabetes mellitus (DM), can have a negative impact on the hypothalamus-pituitary-testis axis (HPT axis), resulting in reduced secretion of gonadotropins and the production of total testosterone (TT), combined with impaired functioning of endothelial cells. Ultimately, this sequence of events can lead to hypogonadism, testosterone insufficiency, and reduced sensitivity of androgen receptors, resulting in erectile dysfunction in individuals (56, 57). Furthermore, this sequence of events results in decreased vascular NOS production, reduced expression and function of PDE5, and inhibited release of nNOS by neurons. Consequently, this leads to a reduction in cGMP levels, causing conditions of smooth muscle relaxation and irregularities in the NO/cGMP signaling pathway (58, 59). A meta-analysis of testosterone treatment for T2DMED revealed that individuals with ED had a greater duration of DM, a higher body mass index (BMI), and lower blood testosterone levels in comparison to those without ED. The duration of diabetes mellitus and serum testosterone levels were found to be independent predictors of erectile dysfunction in logistic regression analysis (60). When the total testosterone level (TT) was ≤ 8nmol/L, the application of undecanoate testosterone significantly improved sexual function in DMED patients (61).

### Tunica albuginea fibrosis

Persistent high blood sugar levels in the setting of diabetes can trigger the restructuring of the extracellular matrix, finally promoting fibrosis. A significant portion of individuals with diabetes suffer from fibroproliferative illnesses, particularly those that cause the formation of fibrotic plaques in the tunica albuginea, a feature of Peyronie’s degenerative disease (PD). Penile dystrophy (PD) increases the likelihood of significant penile abnormalities and disruptions in penile blood circulation, therefore increasing the vulnerability to erectile dysfunction (62, 63). A study conducted in Iran in 2019 revealed a prevalence of PD amounting to 3.8% among a cohort of 317 men diagnosed with type 2 diabetes mellitus (T2DM) (64). In a separate retrospective analysis encompassing 1622 male individuals seeking care at a urological health center, a significant association was established between the presence of T2DM (n = 387; 24%) and PD (*P* = 0.005) (65).

## Psychosocial factors

Diabetes is a metabolic condition requiring lifelong treatment that can result in various complications, placing significant psychological and financial burdens on patients. Emotional instability can significantly impact the erectile function of individuals with diabetes, potentially inhibiting normal penile erection (66). Studies suggest a correlation between sexual function and social, psychological, and organic factors (67). Individuals with diabetic erectile dysfunction often experience heightened states of depression and anxiety, with Nesfatin-1 identified as a potential biomarker for assessing anxiety severity. Those with low serum Nesfatin-1 levels, particularly, must receive psychiatric care (68, 69). In summary, diabetes is a chronic, systemic, and socially impactful disease that affects both physical and mental well-being. Individuals with diabetic erectile dysfunction are particularly prone to mental and psychological challenges.

## Modern medical treatment approaches for diabetes mellitus erectile dysfunction

**Figure 4:**
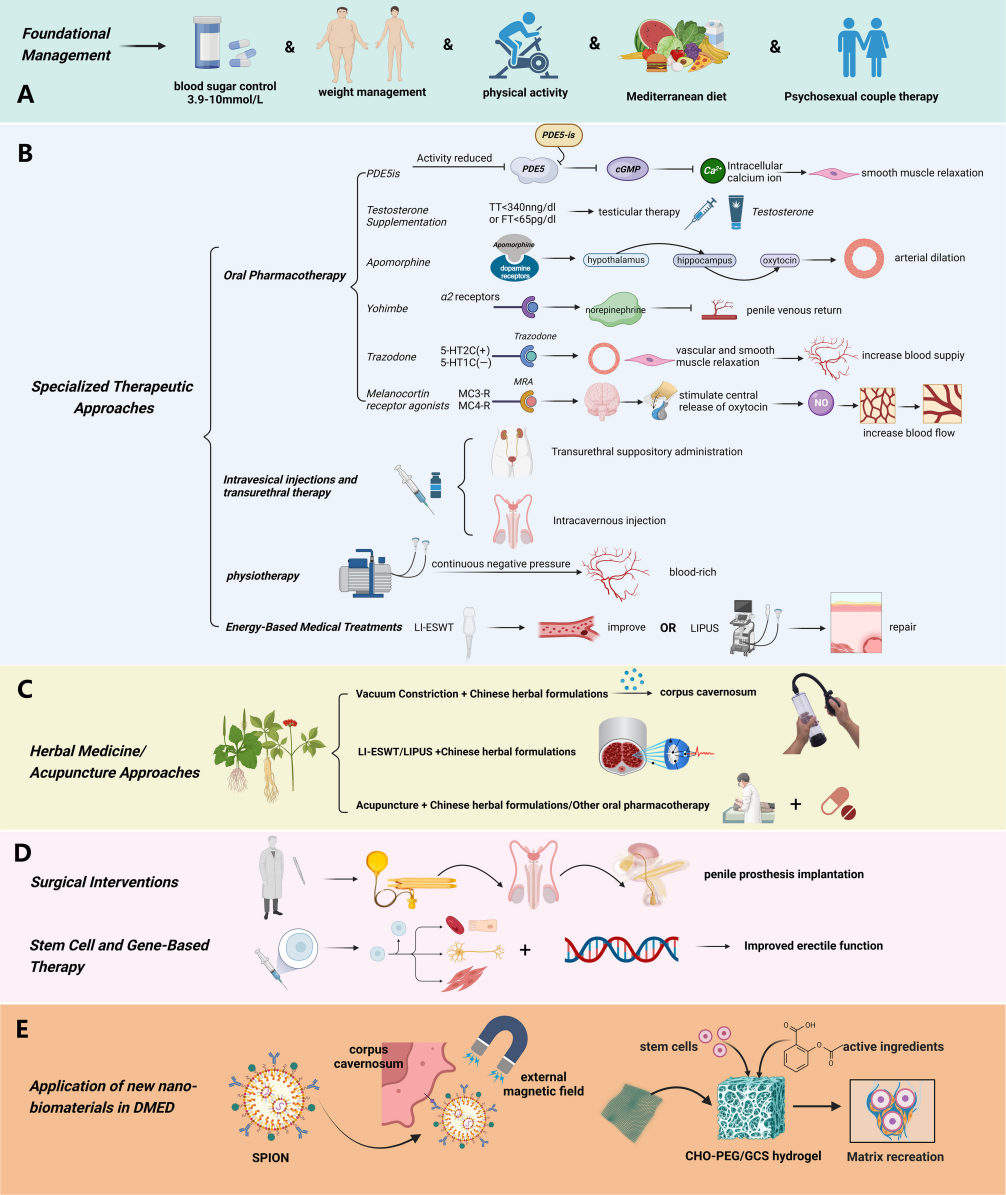
Treatment Strategies of diabetic erectile dysfunction. **(A)** The basic management plan for diabetic erectile dysfunction mainly includes controlling blood sugar in a reasonable range, weight management, and lifestyle adjustment (proper exercise, Mediterranean diet, etc.); **(B)** Specialized treatment of diabetic erectile dysfunction mainly includes: oral drug treatment (PDE5is, Testosterone, Apomorphine, Yohimbe, Trazodone), Intracavernosal Injection and Transitional Suppository Therapy, Physiotherapy, Energy Medical Treatments; **(C)** The comprehensive therapies based on natural drugs mainly include: combination of Chinese herbal medicine with PDE5 inhibitors, Chinese herbal formulas for cavernous body soaking and vacuum construction device, combination of Chinese herbal medicine with extracorporeal shock wave therapy, acupuncture, moxibustion, and acupoint injection with first line drugs and other physical therapied; **(D)** Surgical Interventions and Stem Cell and Gene-Based Therapy. (Used Abbreviations and symbols in the figure: *PDE5is*, Phosphodiesterase Type 5 Inhibitors; cGMP, guanosine monophosphate; TT, total testosterone; LI-ESWT, Low-intensity Extracorporeal Shockwave Therapy; LIPUS, low-intensity pulsed ultrasound; **(E)** SPION, Superparamagnetic Iron Oxide Nanoparticle; CHO-PEG/GCS hydrogel, benzaldehyde terminated poly (ethylene glycol)/glycol chitosan hydrogel) Figure created by BioRender.com.

Currently, there is no clear globally validated treatment regimen for DMED. Nevertheless, based on the current body of research and widely accepted recommendations, the optimal treatment strategy entails a combination of basic care and specialized intervention, emphasizing the proactive control of blood sugar levels from the beginning of the illness. This technique requires developing individualized tactics that correspond to the fundamental processes, with the particular therapeutic interventions differing to some extent depending on the clinician’s level of knowledge. (Shown in **Figure 4**).

### Foundational management

Enhancing blood sugar control stands as the central objective in ameliorating DMED, necessitating consideration of factors such as age of onset and patient body weight. According to guidelines from the American Academy of Clinical Endocrinologists (AACE) on the utilization of novel diabetes management technologies (70), for individuals with general diabetes, a target blood sugar range of 3.9 to 10 mmol/L is recommended. For elderly individuals or those at heightened risk of diabetes-related complications, prioritizing elevated blood sugar control over excessively low levels is crucial to mitigate the risks of hypoglycemia. Hemoglobin A1c (HbA1c) levels ranging from 6.5% to 7.5% warrant monotherapy, with metformin serving as the preferred initial option. Within the HbA1c range of 7.5% to 9.0%, combination therapy with two agents is typically indicated, with the second agent encompassing either oral or injectable hypoglycemic agents. In cases where HbA1c exceeds 9.0%, a combination of 2 to 3 hypoglycemic agents can be considered, and in instances of notable weight loss, the incorporation of at least one insulin preparation is advised. Since overweight and obesity are independent risk factors for the onset of type 2 diabetes, early weight management is particularly important. According to consensus recommendations on weight management, individualized weight loss is recommended, with weight loss of 5% -15% or more as the weight management goal for T2DM patients (71). In addition, for patients with DM complicated with clear vascular and neuropathy, routine treatment with antioxidants, neurotrophic drugs, and medication to improve microcirculation can be given (72–76). Psychosexual couple therapy is also a fundamental guideline in the treatment of erectile dysfunction (ED). Numerous surveys and experiments have highlighted the indispensable role of the sexual partner’s involvement in medical interventions for ED (77), this often manifests in the psychological and emotional encouragement provided by the partner, as well as their contribution to treatment decision-making. Additionally, the partner plays a crucial role in achieving long-term remission of ED after treatment, with their attitudes, beliefs, and sexual experiences also being significant to the prognosis of ED (78).

Lifestyle modifications exert a favorable impact on blood sugar regulation and enhancement of erectile function. Research substantiates that judicious physical activity imparts a safeguarding effect on male erectile function, thereby diminishing the incidence of DMED (79, 80). Among patients afflicted with DMED, an 8-week exercise regimen involving 45-60 minutes of daily activity exhibited an ameliorative effect on erectile function, in contrast to sedentary controls (81). Multiple antecedent investigations have yielded findings indicative of the Mediterranean diet’s efficacy in safeguarding erectile function. Moreover, antihyperglycemic agents seem to confer a comprehensive safeguarding influence on erectile function (82).

### Specialized therapeutic approaches

DMED patients necessitate specialized intervention for erectile concerns in conjunction with routine blood sugar management and lifestyle modifications, with the aim of expeditiously rectifying erectile dysfunction.

#### Oral pharmacotherapy

##### Phosphodiesterase type 5 inhibitors

PDE5is serves as the frontline therapeutic option for erectile dysfunction. These agents foster and sustain erection by obstructing the hydrolysis of cGMP within cavernous smooth muscle via the enzyme PDE5is. This interference extends the duration of cGMP activity, thereby curtailing intracellular calcium levels and preserving smooth muscle relaxation, consequently sustaining penile erection. Presently, several PDE5 inhibitors are available within the Chinese market, encompassing sildenafil, tadalafil, vardenafil, and avanafil. While their therapeutic efficacy is akin, disparities exist in terms of onset and metabolism times. During the medication process, it is important to avoid the concurrent use of nitrate drugs (83) and potent inhibitors of CYP3A4 to prevent an increase in the plasma concentration of phosphodiesterase type 5 inhibitors (PDE5is) (84).

##### Testosterone supplementation

Testosterone supplementation yields notable efficacy in cases of DMED coupled with androgen deficiency or insufficiency. Presently, collaborative recommendations from various institutions suggest that mild testosterone deficiency can be identified when serum total testosterone (TT) falls below 340 ng/dl, while severe deficiency is indicated at TT levels below 230 ng/dl, and the administration of testosterone supplementation to patients yields discernible therapeutic benefit (85, 86). Furthermore, the correlation between age, blood sugar level, and free testosterone (FT) is substantial, underscoring the potential value of supporting testosterone supplementation therapy when FT levels dip below 65 pg/ml (87, 88). A meta-analysis encompassing 16 pertinent studies demonstrated that hypogonadal men subjected to testicular therapy exhibited a notably higher rate of erectile dysfunction improvement relative to those receiving placebo (57.0% versus 16.7%) (89).

##### Apomorphine

Apomorphine functions as an agonist for dopamine D2 receptors. Its mechanism involves stimulating dopamine receptors located in the paraventricular nucleus, consequently engendering activation of the hypothalamus-hippocampus-oxytocin pathway. This signaling cascade is transmitted through the spinal cord to the penis, culminating in arterial dilation within the penile region and engendering heightened blood circulation. This augmented blood flow fosters erection. The drug received approval from the European Medicines Agency in February 2001 for the management of ED, and select studies have demonstrated its safety and efficacy in treating ED patients (90). During the administration, it is crucial to avoid the combination with 5HT3 antagonists to prevent the occurrence of severe adverse reactions such as hypotension, loss of consciousness, or coma (91).

##### Yohimbe

Yohimbine selectively antagonizes presynaptic α2 receptors, facilitating the liberation of norepinephrine. This phenomenon augments the discharge of norepinephrine from nerve endings within the cavernous body, diminishing the penile venous return and fostering a state of engorged erection (92). Prior to the advent of PDE5 inhibitors in the treatment of ED, yohimbine was extensively employed for this purpose; however, its efficacy and safety profile have not undergone comprehensive evaluation. Presently, its use has waned. During the use of certain medications, it is important to avoid co-administration with monoamine oxidase inhibitors (MAOIs), such as phenelzine (Parnate) and other antidepressant drugs, to prevent potentially serious adverse reactions (93).

##### Trazodone

Trazodone functions as an agonist for serotonin 2C receptors (5-HT2C) and an antagonist for 5-HT1A receptors. Besides its central nervous system effects, the drug can also exert alpha2 receptor blockade. Its potential mechanism of action entails α2 receptor blockade, fostering vascular and corpus cavernosum smooth muscle relaxation, thereby augmenting blood supply to the penile corpus cavernosum and facilitating erection. While clinical reports have attested to trazodone’s effectiveness in treating ED, results from meta-analyses suggest no statistically significant difference when compared to placebo (94). When administering medication, special consideration should be given to patients with erectile dysfunction (ED) who have severe heart disease or arrhythmias, as the use of such medications may be contraindicated or require caution in these individuals (95, 96).

##### Melanocortin receptor agonists

The melanocortin system regulates sexual function by activating melanocortin receptors (MCRs), particularly MC3-R and MC4-R, which are distributed in the hypothalamus and spinal cord. The activation of these receptors stimulates the central and spinal release of oxytocin, which in turn increases the production of nitric oxide (NO), leading to the relaxation and engorgement of the penile erectile tissue, thus facilitating erection. Melanocortin receptor agonists (MRA), such as Melanotan II (MT II) and Bremelanotide (PT-141), mimic this natural process, and they have shown potential in the treatment of male erectile dysfunction (ED) in clinical studies (97). These drugs can directly act on the brain’s sexual behavior control centers, as well as increase penile blood flow by promoting NO production, helping men achieve and maintain erections with or without sexual stimulation. This mechanism differs from that of PDE5 inhibitors, which require sexual stimulation to facilitate erections, whereas MRAs can trigger erections without sexual stimulation, offering a novel pharmacological approach to ED treatment. The usage of the above drugs and the intervention course are detailed in **Table 1**.

**Table 1:** Oral medications for DMED: administration methods, dosage, and treatment duration.

Reference	Drug or	Administration method	Dosage	Course of treatment
Venneri MA et al., 2019 (135)	*Sildenafil( PDE5is)*	Take orally	The prescribed doses are 25, 50, and 100mg, with a recommended starting dose of 50mg	Take once a day or every other day, each effect can maintain 12h
Melehan KL et al., 2018 (136) Tan HM et al., 2008 (137)	*Vardenafil( PDE5is)*	Take orally	Once a day, depending on the efficacy and tolerability, the dose can be adjusted to 5mg or 20mg. The maximum recommended dose is 20mg a day	The effect takes 15 to 30 minutes
Cui H et al., 2015 (138)	*Tadalafil( PDE5is)*	Take orally	10-20mg	It starts in about 30 minutes and can last up to 36 hours
Mulhall, J P et al., 2001 (139)	*Apomorphine*	Sublingual ingestion	Start with 2 mg and adjust to a maximum of 4 mg as needed	Screening period: 2 weeks to determine baseline severity of ED. Dose optimization period: 3 weeks, starting at 2 mg, adjusting the dose as needed. Treatment duration: 4 weeks, patients take their individualized optimal dose.
Bloomer RJ et al., 2015 (140) Lee SR et al., 2013 (141)	*Yohimbe*	Take orally/Subcutaneous injection	Oral: 5-10mg 3 times a day Subcutaneous injection: 10-20mg each time, 2-3 times a day	Oral: 10 weeks Subcutaneous injection: 20 times
Goyal P et al., 2023 (142) Hadi F et al., 2022 (143)	*Trazodone*	Take orally	The recommended initial dose is 50 to 100mg/day (divided dose), the dose can be increased by 50mg/day every three to four days, and the maximum dosage is not more than 400mg/day (divided dose).	The best results appear two weeks to four weeks after taking the drug
Ückert, Stefan et al., 2014 (97)	*MT II( Melanocortin receptor agonists)*	Subcutaneous injection	0.025 mg/kg	Courses of treatment in clinical studies vary, with some designed to be monitored for hours after a single dose, while others are administered multiple times over several weeks.
Ückert, Stefan et al., 2014 (97)	*PT-141( Melanocortin receptor agonists)*	Intranasal administration	7 mg-15 mg	Courses of treatment in clinical studies vary, with some designed to be monitored for hours after a single dose, while others are administered multiple times over several weeks.

##### Intracavernosal injection and transurethral suppository therapy

Secondary methods of treating erectile dysfunction include injecting the patient intravenously or administering a suppository through the urinary tract. Their main benefit is that they help speed up the process of getting an erection by allowing the medicine to be absorbed locally and start working quickly. Commonly used drugs include vasoactive intestinal polypeptide, phentolamine, papaverine, and alprostadil. Once they’ve gotten the proper training, males can administer cavernosal injections on their own or with the help of a partner. Localized massage can speed up the erection process, which may have nothing to do with sexual desire, and the erection usually appears within 10 minutes after administration (98). Presently, these approaches find application in the evaluation and diagnosis of ED in China. Nonetheless, intracavernosal self-administration is a practice adopted by only a minuscule proportion, and topical application might precipitate priapism, albuginea fibrosis, and symptoms like penile, urethral pain, or burning sensation (99).

##### Physiotherapy

Using an elastic band fastened at the base of the penis, the Vacuum Constriction Device (VCD) causes erection by applying constant negative pressure to the penis, which helps to fill the cavernous body with blood and maintain blood perfusion. Notwithstanding the device’s simplicity of use, the erection produced by vacuum suction is described as artificial and mechanical, frequently accompanied by a cold feeling in the treated region. A significant percentage, roughly 50%, of patients indicate discontent with this method (100). VCD is deemed appropriate for individuals who have encountered failure with oral PDE5is therapy and harbor aversion to other, more invasive interventions. Side effects attributed to vacuum suction encompass localized ecchymosis, penile numbness, and delayed ejaculation (101).

##### Energy-based medical treatments

The main application of Low-intensity Extracorporeal Shockwave Therapy (LI-ESWT) is to treat moderate vascular erectile dysfunction (ED), which is often linked to impaired cavernous blood arteries and endothelial cell dysfunction in patients with DMED. Therefore, it may be employed either as an independent method or in combination with other pharmacological treatments. Existing research suggests that LI-ESWT can improve the IIEF score and erection hardness score in patients with mild vascular erectile dysfunction. Furthermore, the use of LI-ESWT medication may enhance the effectiveness of PDE5 inhibitors in patients who show insufficient response to first PDE5 inhibitor therapy (102). The clinical investigations indicate that LI-ESWT is a safe and effective therapy for men with well-controlled DM with moderate or improved erectile dysfunction. However, the longevity of the benefit is lower in diabetic men compared to nondiabetic men. Further monitoring is required to assess the therapeutic effect on DMED patients (103, 104).

Low-intensity pulsed ultrasonography (LIPUS) has the capacity to promote tissue healing, accelerate the regeneration of soft tissues, and reduce tissue inflammation (105). Research findings suggest that LIPUS therapy improves erectile function in diabetic rats, corrects abnormalities in penile tissue, increases intracavernous pressure (ICP) levels, increases endothelial and smooth muscle content, increases the expression of eNOS and nNOS, causes changes in collagen and fiber composition, and decreases the TGF-β1/Smad/CTGF signaling pathway (106). One study, a multicenter double-blind Randomized Controlled Trial (RCT), showed that LIPUS significantly improved the IIEF5 score in patients with mild to moderate erectile dysfunction (107). While its underlying molecular mechanism requires further exploration, LIPUS shows potential as a therapy for erectile dysfunction.

##### Surgical interventions

Surgical procedures are a third-tier method for treating erectile dysfunction. In an increasing number of individuals with DMED who do not respond to medication, penile prosthesis implantation is being considered as a final option. Nevertheless, the surgical modification of cavernous tissue during this operation makes it impossible to achieve future restoration of smooth muscle relaxation. At both national and international levels, the hydraulic three-piece implant is widely regarded as the preferred option for penile prosthesis. Significantly, the satisfaction percentages among the beneficiaries and their partners are very high, accounting for 70% and 90% respectively (108). Infection is a common consequence linked to penile prosthesis, with a prevalence rate of 2%–4% in cases (109). Additional surgical procedures for erectile dysfunction include artery bypass surgery and vein ligation to treat congenital aberrant venous leakage. Nevertheless, vascular surgery is rarely performed by andrologists today because to the questionable effectiveness advantages (110).

### Herbal medicine approaches

Traditional Chinese herbal therapy has a well-established history and proven therapeutic effectiveness in the treatment of ED. The integration of Chinese herbal compounds, which have functions such as improving blood circulation, reducing blood stasis, and unblocking meridians, as well as tonifying the kidney, harmonizing the liver, and boosting vital energy and nurturing yin, in combination with oral PDE5 inhibitor administration, has been shown to not only enhance therapeutic outcomes but also exemplify the holistic regulation characteristic of traditional Chinese medicine. Moreover, this method can interfere in fundamental genetic disorders, providing a viable therapeutic approach for reducing erectile dysfunction issues in early-stage diabetic patients. Furthermore, the use of Chinese herbal preparations for treating DMED by cavernous body immersion and vacuum constriction device is a fascinating combination of traditional Chinese and Western medicine. This approach exploits the absorption of medication ingredients to enhance the process of cavernous body repair (111, 112). The combination of traditional Chinese herbal medicine with extracorporeal shock wave therapy has been shown to have a significant synergistic impact in improving erectile function in diabetic erectile dysfunction rats. The aforementioned method not only broadens the scope of clinical treatment options (113), but also emphasizes the potential of integrating acupuncture, moxibustion, and acupoint injection with primary medications and other physical therapies to improve the overall effectiveness in controlling DMED (114, 115).

### Stem cell and gene-based therapy

Stem cells, also known as SCs, are distinguished by their ability to differentiate in multiple directions or in a specific one, as well as by their potential to continue self-renewal. They possess substantial utility in the fields of regenerative medicine and tissue engineering. With the development of regenerative medicine ideas and the evidence of successful animal trials, SCs have attracted significant attention as a possible treatment for male erectile dysfunction (116). In their study, Yang et al. (117) introduced stem cells generated from adipose tissue and endothelial progenitor cells into a rat model of DMED. Their findings revealed a significant enhancement in erectile function among the group treated with stem cells as compared to the group treated with DMED four weeks after injection. The effectiveness was much higher when the two types of cells were combined. Moreover, numerous studies have examined the synergistic effects of stem cells used in combination with traditional therapies. One example is the work undertaken by Shan et al. (118) which involved the use of bone marrow mesenchymal stem cells in conjunction with low-energy shock waves in DMED rats. It was shown that the combination method exhibited superior performance compared to the individual use of stem cells or low-energy shock waves. The study conducted by Bahk et al. (119) included seven patients who were unresponsive to medical treatment and were in need of prosthetic surgery. A dose of 1.5 × 1.7 times the standard of human cord blood cells was administered to these individuals via injection into the corpus cavernosum. Multiple factors were recorded in the trial, including IIEF-5 scores, intercourse profiles, blood glucose diaries, and medication dosages. After careful consideration, only two patients chose to have penile prosthesis implantation, while the other patients successfully restored erectile function and achieved good sexual intercourse.

In brief, although stem cell therapy has shown efficacy in many animal models of erectile dysfunction (ED), there is a scarcity of clinical trial studies on DMED, and several unresolved issues still needs to be tackled. For example, the long-term efficacy and safety of the treatment have not been confirmed, and the standardization of stem cell therapy is insufficient because of significant differences in stem cell synthesis methodology. Furthermore, ethical considerations are also relevant. However, stem cells maintain substantial therapeutic potential, and ongoing investigation of their ability to treat DMED remains encouraging.

Gene therapy is a novel therapeutic method that specifically aims to introduce external genetic material into cells in order to correct or compensate for faulty genes (120). As our understanding of the molecular processes responsible for the pathophysiology of DMED improves, more identified treatment targets are consistently being revealed. A multitude of genetic approaches have been subjected to thorough tests and shown effective in diverse animal models. These approaches mostly involve preclinical and limited clinical studies, which concentrate on cellular targets and various viral vectors/nanoparticles for delivering genes. The majority of gene therapy research conducted on DMED have utilized three primary viral vectors, namely adenovirus, AAV, and HSV, which contain genomes that remain outside the chromosomes within the nucleus. Experimental results suggest that gene therapies including synthetic nitric oxide synthase (NOS) (121, 122), neurotrophic factors (123–125), angiogenic growth factors (126, 127), Ca^2+^-sensitive maxi-K^+^ Channels (128), antifibrotic factors (129), and vasoactive intestinal polypeptide (VIP) (130) may play crucial roles in promoting relaxation of cavernous smooth muscles, preventing fibrosis in cavernous tissue, improving vascular endothelial function, and repairing nerve damage in living organisms. Therefore, these treatments have the capacity to improve erectile function in DMED model organisms.

Hence, it is evident that gene therapy holds the promise to address DMED and diminish patient reliance on PDE5 inhibitors. Moreover, the amalgamation of gene therapy with stem cell therapy could potentially rejuvenate spongy tissue architecture. Leveraging the multilineage potential and limited immunogenicity of stem cells, this combination may facilitate prolonged *in vivo* target gene expression. Consequently, a notable enhancement in erectile function has been observed in animal models through combination therapy, surpassing the outcomes of stem cell therapy in isolation. Notwithstanding, the present-day application of gene therapy for DMED remains in its formative stages. Although preclinical studies have identified multiple molecular targets with potential for preventing and treating DMED, the lack of sufficient clinical investigation hampers the validation of their applicability. Moreover, existing studies predominantly emphasize therapeutic outcomes, often sidelining varied effects. Additionally, the use of naked DNA and plasmids aims to attenuate immunogenic responses and cytotoxicity. However, their transient gene expression *in vivo* curtails their ability to engender enduring therapeutic effects in ED treatment.

### Application of new nano-biomaterials in DMED

Nanobiomaterials are under increasing development for addressing challenges in DMED treatment, including low oral drug bioavailability, insufficient targeting of organs, systemic adverse reactions, and the stability of stem cell/gene therapy. Carrier materials possessing high biocompatibility, low immunogenicity, active targeting, and low invasiveness are employed to enhance the targeting and efficacy of intervention methods involving stem cells, growth factors, bioactive proteins, and drugs. Some experimental studies have confirmed the effectiveness of these approaches in improving DMED. For instance, Zhu LL et al. (131) transfected superparamagnetic iron oxide nanoparticles (SPION) into rat adipose-derived stem cells (ADSCs), creating SPION-ADSCs. These were then injected into the streptozotocin-induced DMED rat penis sponge, and relevant tests were conducted four weeks later. The results demonstrated that the application of an external magnetic field enhanced the homing efficiency of SPION-ADSCs to the corpus cavernosum area, leading to increased smooth muscle and endothelial density in the cavernous body, thereby improving erectile function. Han G et al. (132)developed curcumin-loaded silane-based hydrogel nanoparticles and applied them to the abdominal skin of DMED rats until complete absorption, achieving local non-invasive administration, sustained release, reduced medication frequency, and avoidance of gastrointestinal adverse reactions. This approach significantly improved drug efficiency, benefiting DMED treatment. Lu, et al (133), discovered that the benzaldehyde terminated poly (ethylene glycol)/glycol chitosan (CHO-PEG/GCS) hydrogel is a promising stem cell carrier that enhances adipose stem cells and ameliorates diabetes-induced CD31 fibrosis, apoptosis of positive endothelial cells, α-SMA positive smooth muscle, and NeuN positive nerve fibers. Draganski, Andrew, et al. (134) discovered that topical application of curcumin-loaded nanoparticles (curc-NP) to a T2DM rat model can achieve systemic delivery of curcumin, treating erectile dysfunction (ED) and regulating the body’s expression of inflammatory markers.

Present research on carrier materials primarily concentrates on substances like hydrogels with diverse characteristics, fat-soluble particles, and magnetic particles. These materials can serve as sustained-release stents for local body intervention, enhance drug lipophilicity, or act as scaffolds for various cytokines, stem cells, and drugs. The carrier functions as a supportive structure, offering sustained/controlled release in the target area, thereby enhancing the efficacy of the initial treatment method and minimizing side effects from the intervention. Nevertheless, current research on the utilization of carrier materials in DMED treatment remains confined to animal experiments. Further discussions are necessary regarding the implementation of clinical trials and the promotion of application, all while ensuring safety.

## Limitations

In conducting this review on DMED, it’s essential to recognize the inherent limitations. Firstly, our review relies heavily on existing literature and clinical studies, which may have their own biases and methodological limitations. These studies often vary in terms of sample size, design, and duration, which can introduce heterogeneity into the data. Additionally, publication bias may exist, as studies with positive results are more likely to be published than those with negative findings, potentially skewing our analysis. Secondly, while we have attempted to provide a comprehensive overview, the field of DMED research is vast and continually evolving. New studies and treatments may have emerged after our review’s cutoff date, which could affect the completeness and timeliness of our analysis. Lastly, the quality of evidence in some areas, such as the use of traditional Chinese herbal medicine, is not as robust as that in more conventional medical approaches. This limitation highlights the need for more rigorous research in complementary therapies for DMED.

## Conclusion and future perspectives

In conclusion, this review provides a synthesized perspective on the complex landscape of DMED based on existing literature and clinical studies. While we have discussed various physiological and pathological mechanisms underlying DMED and outlined a diverse range of treatment options, it is crucial to acknowledge the limitations of our analysis. The reliance on existing research means that our conclusions are contingent on the quality and comprehensiveness of the studies available. Moreover, the rapidly evolving nature of DMED research necessitates ongoing updates and revisions to our understanding and recommendations. Despite these limitations, our review underscores the multifaceted nature of DMED and the need for a holistic approach that combines pharmacological, lifestyle, and alternative therapies to effectively manage this condition.

Beyond the present scope, the DMED research landscape offers a canvas abundant with promise and unexplored avenues. Navigating the complexities of DMED and delving deeper into its enigmatic mechanisms underscore that our journey is far from concluded. Unraveling these complexities and formulating pioneering interventions is now more imperative than before. In the domain of energy medicine, a paradigm-shifting frontier beckons, providing potential solutions that harness the body’s innate healing energies. Investigating the interplay among energy pathways, nerve signaling, and erectile function may reveal novel strategies to mitigate DMED.

Furthermore, the integration of traditional herbal medicine with modern medical practices and other complementary therapies presents an intriguing trajectory for future exploration. This pathway gains significance, especially in light of the historical use of holistic methods in tackling sexual dysfunction. The synergy between ancient wisdom and modern medical advancements holds the potential to provide distinctive insights and innovative treatment directions.

Concurrently, the regenerative potential of stem cells remains a focal point in the realm of therapeutic opportunities. Collaborative efforts spanning stem cell research and DMED may herald breakthroughs that redefine treatment efficacy boundaries. Gene therapy, capable of intervening at the core of molecular dysfunction, introduces a new layer of promise. Tailored genetic interventions could potentially rectify the aberrant pathways underlying DMED. Merging gene therapy with stem cell approaches has the potential to generate a synergy surpassing the constraints of individual approaches, resulting in enduring and impactful therapeutic outcomes.

While progressing, a comprehensive and integrative approach becomes of utmost importance. Integrating traditional herbal medicine, energy medicine, stem cells, and gene therapy with established modalities might yield a diverse array of solutions addressing DMED’s multifaceted nature; using novel biomaterials as vehicles to improve the feasibility and effectiveness of pharmaceutical, stem cell, and gene therapies is worthy of in-depth study.

However, the path ahead is not devoid of challenges. Ethical considerations, safety profiles, and the translation of laboratory findings to clinical efficacy continue to be subjects of close examination. Rigorous experimentation, robust clinical trials, and transparent discussion are imperative for validating and refining these emerging therapeutic paths.

In conclusion, the field of DMED research holds immense potential for further exploration in areas such as biochemistry, traditional medicine, energy medicine, stem cells, and gene therapy. Integrating ancient wisdom and modern science in research methods has shown promising results in the pursuit of revolutionary breakthroughs for DMED treatment. With the continuous efforts of scientists from around the world, we firmly believe that the prevention and control strategy of DMED will be redefined.
